# Impact of externally facilitated continuous quality improvement cohorts on Advanced Access to support primary healthcare teams: protocol for a quasi-randomized cluster trial

**DOI:** 10.1186/s12875-023-02048-y

**Published:** 2023-04-11

**Authors:** Mylaine Breton, Isabelle Gaboury, Elisabeth Martin, Michael E. Green, Tara Kiran, Maude Laberge, Janusz Kaczorowski, Noah Ivers, Nadia Deville-Stoetzel, Francois Bordeleau, Christine Beaulieu, Sarah Descoteaux

**Affiliations:** 1 Université de Sherbrooke, Campus Longueuil, 150 Place Charles-LeMoyne, Office 200, Longueuil, QC J4K 0A8 Canada; 2 Queen’s University, Kingston, ON Canada; 3 University of Toronto, Toronto, ON Canada; 4 Université Laval, Québec, Québec Canada; 5 Université de Montréal, Montréal, Québec Canada

**Keywords:** Advanced Access, Primary care, Continuous quality improvement, Audit and feedback, Interprofessional care

## Abstract

**Background:**

Improving access to primary health care is among top priorities for many countries. Advanced Access (AA) is one of the most recommended models to improve timely access to care. Over the past 15 years, the AA model has been implemented in Canada, but the implementation of AA varies substantially among providers and clinics. Continuous quality improvement (CQI) approaches can be used to promote organizational change like AA implementation. While CQI fosters the adoption of evidence-based practices, knowledge gaps remain, about the mechanisms by which QI happens and the sustainability of the results. The general aim of the study is to analyse the implementation and effects of CQI cohorts on AA for primary care clinics. Specific objectives are: 1) Analyse the process of implementing CQI cohorts to support PHC clinics in their improvement of AA. 2) Document and compare structural organisational changes and processes of care with respect to AA within study groups (intervention and control). 3) Assess the effectiveness of CQI cohorts on AA outcomes. 4) Appreciate the sustainability of the intervention for AA processes, organisational changes and outcomes.

**Methods:**

Cluster-controlled trial allowing for a comprehensive and rigorous evaluation of the proposed intervention 48 multidisciplinary primary care clinics will be recruited to participate. 24 Clinics from the intervention regions will receive the CQI intervention for 18 months including three activities carried out iteratively until the clinic’s improvement objectives are achieved: 1) reflective sessions and problem priorisation; 2) plan-do-study-act cycles; and 3) group mentoring. Clinics located in the control regions will receive an audit-feedback report on access. Complementary qualitative and quantitative data reflecting the quintuple aim will be collected over a period of 36 months.

**Results:**

This research will contribute to filling the gap in the generalizability of CQI interventions and accelerate the spread of effective AA improvement strategies while strengthening local QI culture within clinics. This research will have a direct impact on patients’ experiences of care.

**Conclusion:**

This mixed-method approach offers a unique opportunity to contribute to the scientific literature on large-scale CQI cohorts to improve AA in primary care teams and to better understand the processes of CQI.

**Trial registration:**

Clinical Trials: NCT05715151.

## Background

### Timely access in primary healthcare

Access is one of the major concerns faced by health systems worldwide. Access to health services is a high priority for the population, clinicians and decision-makers alike [1]. Timely access, such that patients can access the care they need when they need it, is one of the cornerstones of strong primary healthcare (PHC). Across Canada, timely access remains a major challenge. A recent international report documenting primary care access found that Canada ranks poorly compared to other high-income countries. According to a 2020 report by the Commonwealth Fund, only 41% of Canadians reported being able to get a same or next day appointment to see a doctor or a nurse the last time they needed medical attention [2]. This is a 5% decline since the previous report in 2016 [2]. Limited access increases the risk of poor health outcomes and health disparities [3, 4] and increases costs for the healthcare system.

### Advanced Access as a solution to improve access and continuity of care

Of the various organisational innovations developed to improve timely access to care, Advanced Access (AA) is one of the most recommended models [5]. Rooted in patients’ relational and informational continuity with a PHC professional/team to increase accessibility, the AA model ensures that patients obtain timely services based on their needs [6]. Originally developed in the United States in 2001, the effectiveness of the AA model, practiced among physicians and nurses, has been demonstrated in various healthcare systems [7–11]. Benefits of AA include reduced wait times [7, 9, 11–13] and missed appointments [7, 13] and improved professional and patient satisfaction [11, 14] as well as provider productivity [11].

**Fig. 1:**
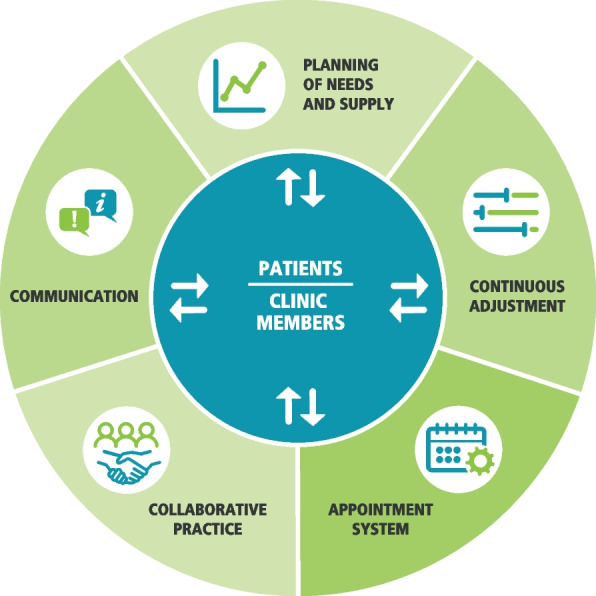
Revised Advanced Access model

Over the last two decades, AA has become increasingly popular in Canada. The model has been widely promoted by the College of Family Physicians of Canada and several other provincial organisations and professional associations [6]. Since the inception of the AA model 20 years ago, our research team has developed a revised model based on a more interdisciplinary team practice through a process of multiple consultations with 45 experts. The revised model is based on five key pillars: 1) comprehensive planning for needs, supply and recurring variations; 2) regular adjustment of supply to demand; 3) processes of appointment booking and scheduling; 4) integration and optimisation of collaborative practice; and 5) communication about AA and its functionalities [15]. The Fig. 1 presented the revised Advanced Access model.

Over the past years, the AA model has been implemented in Canada to varying degrees. On a more local level, the implementation of AA varies quite a lot among both providers and clinics [16, 17]. More importantly, our experience in the field shows that the model has not kept up with the development of PHC team-based approaches or with technological innovations [18]. Indeed, very few other PHC providers, such as social workers, psychologists and pharmacists, have implemented the model, despite its interprofessional scope. With the exception of an ongoing proof of concept led by our research team [19], the literature is scarce on strategies to expand the AA model to the entire PHC team.

### Continuous quality improvement (CQI) approaches

CQI approaches promote organisational change and are often based on improving specific processes, either by eliminating waste from the system (e.g. LEAN) or reducing variability and errors in the process (e.g. Six-Sigma). A widely used approach, the Model for Improvement [20], is designed to promote, structure and sustain changes in organisations to improve both processes and outcomes [21]. It aims to better understand the system itself, such as cultural or structural changes within the organisation, in order to implement a new model or new processes [22]. Following an investigation of a given problem, changes are implemented through iterative implementation cycles of four steps (represented by the acronym PDSA), [20] where change is: 1) planned based on evidence from data, community feedback and/or stakeholder experience (Plan); 2) carried out while documenting its effects (Do); 3) analysed by measuring the results achieved while comparing them to expected results and appreciating the impact of change (Study); and finally 4) refined, either by maintaining or adjusting actions in future cycles or by expanding its scale (Act).

### Factors and techniques for successful CQI

While CQI fosters the adoption of evidence-based practices [20] and is instrumental to achieve the quintuple aim, multiple strategies have been used to support organisational change [23, 24]. Despite emerging evidence on the influence of the implementation context on the outcomes of CQI [25, 26], such as managerial involvement [27], staff attitudes [27–29], interprofessional collaboration [28], effective communication [28], the presence of an internal champion [27], available resources [28], general QI culture [30], the absence of conflict [27], the QI objective itself [30] and fit with the organisation’s priorities [30], knowledge gaps remain, especially about the ideal team size and composition, optimal levels of patient involvement [31–33], and more importantly, the mechanisms [34–36] by which QI happens.

Continuing education alone or combined with other strategies cannot optimally achieve change without intensive follow-up [37, 38]. Experts internal or external to the organisation must accompany the process. This is called practice facilitation [37]. Practice facilitation is especially useful as a stand-alone intervention, compared to more limited strategies such as academic detailing or audit and feedback [37, 39]. Although practice facilitation can also be used effectively in combination with other strategies, studies have shown that tailoring facilitation interventions to the individual is a determining factor to achieve buy-in from stakeholders and, more generally, to implement changes in local settings [40–43]. Using external change agents in the practice facilitation model also appears to strongly facilitate organisational change, especially for smaller settings and PHC services [40–43]. Other enablers of practice facilitation include sustained interactions between facilitators and practices, appropriate and regular frequency of practice facilitation [44, 45] and patient and partner engagement [40–43]. In addition, support and coaching through multiple cycles [46] over a sufficient length of time (12–18 months) [47] are key to complete improvement cycles and achieve change goals [48].

Beyond practice facilitation, the scientific literature offers poor guidance on the criteria to select appropriate strategies to improve the *process* of CQI [49, 50]. This is largely due to the fact that such processes are often poorly defined in empirical studies and rarely or inadequately evaluated [36, 51–53]. This leaves implementers looking to design successful CQI initiatives with vague recommendations on the importance of supporting clinical teams over time through a personalised and evolutive approach focused on relationship building [36]. It is therefore complex to determine with certainty the causal links between CQI processes and their effects.

Studies showing the effectiveness of CQI also highlight its barriers, namely the complexity of healthcare organisations [25], the absence of structure or resources [25, 54, 55], poor understanding (and use) of CQI components [56] or support that is too succinct [25, 47]. Above all, successful CQI is time-consuming and resource intensive [57, 58]. Group mentoring, defined as ongoing group activities where participants with different levels of experience working in similar fields share their experience, increase their skills and take part in opportunities to reflect on their own practice [59], could accelerate PDSA cycles. This rapid knowledge transfer strategy could contribute to increasing the scale for tests of change and, consequently, the spread of successful implementation strategies [60]. In particular, sharing the optimal sequencing of tasks can help other teams plan their change strategies by building on what works or does not work in other teams [20]. Group mentoring through CQI cohorts could reduce isolation, bring new energy to QI strategies and improve knowledge about what works and what does not [61, 62].

Finally, studies focusing primarily on the sustainability of complex innovations supported through CQI are lacking [63, 64]. The few studies available underline the importance of focusing on the sustainability of the process up front, [65] adaptability of the CQI initiative [66] and relevance of the initiative for the organisation [66]. Identifying processes leading to long-term impacts of CQI is key to develop practical guidelines for healthcare teams who wish to improve patient care.

### Preliminary work: AA and CQI

Between 2008 and 2010, an Ontario-based CQI learning collaborative program (called QIIP) was available to all Family Health Teams, with some teams focusing, among other themes, on the improvement of their practice in AA. QIIP included three 2-day face-to-face learning sessions, action periods and a summative congress [67] offered in three waves occurring over about 15 months. The program failed to show significant differences between the intervention and control groups for most indicators, including the 3rd next available appointment [67]. Qualitative data suggested significant improvements in some practices, but also a high degree of variability among clinics. Of note, QIIP was successful in facilitating interdisciplinary team functioning. The QIIP team concluded that there was a need for further direct evaluation of QI approaches to improve AA in the future [67, 68].

The Quebec team members have developed extensive expertise and experience in assessing the implementation of AA among different PHC providers [69] and support individual reflection through the use of an online reflexive tool and a rigorous CQI process [19]. This intervention has shown an average decrease of 7 days to the 3rd next appointment and a decrease of 11% on the proportion of available time slots within the next 48 h. This project shed light on the fact that strategies to improve AA are often common across PHC teams and that there is a loss of efficiency when each provider and team are supported individually. Thus, we hypothesise that the addition of group mentoring would allow for system gains by allowing teams of providers 1) to have access to the strategies and learnings of other teams that have experienced it and 2) to share in real time with teams experiencing similar challenges.

There is great potential to improve access in PHC by supporting practices interested in improving AA and by expanding the implementation of the model to professionals other than physicians and nurses. The various AA projects conducted so far have 1) shown promising results and 2) contributed to the development of expertise and several tools to assess and guide the implementation of AA for all types of PHC providers. These findings guided our research team in the development and evaluation of a CQI cohort of PHC practices for the improvement of AA.

### Research objectives

**Fig. 2:**
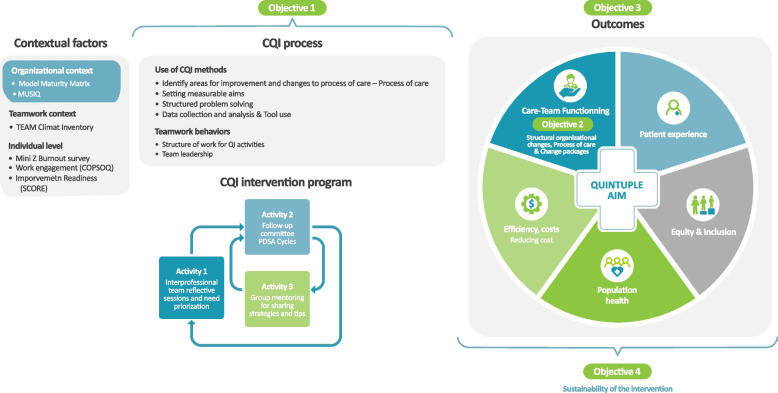
Conceptual framework and research objectives

We propose a multi-method quasi-randomised cluster trial to analyse the implementation and effects of CQI cohorts on AA for PHC clinics (see Fig. 2). Specific objectives are to:

1. Analyse the process of implementing CQI cohorts to support PHC clinics in their improvement of AA.
2. Document and compare structural organisational changes and processes of care with respect to AA within study groups (intervention and control).
3. Assess the effectiveness of CQI cohorts on AA outcomes.
4. Appreciate the sustainability of the intervention for AA processes, organisational changes and outcomes.

### Conceptual framework

The Informing Quality Improvement Research (InQuIRe) framework was chosen to guide this study [70, 71]. This framework is based on a literature synthesis of CQI theories and themes that have been adapted for PHC. The InQuIRe framework outlines (1) contextual factors at the organisational, team and individual levels; (2) the CQI process, including QI methods and teamwork; and (3) outcomes related to structural changes to organisations or processes of care and quality of care. Our hypothesis is that the proposed intervention will have a long-term impact on various AA processes, outcomes and organisational changes and that those will be influenced by contextual factors. The Fig. 2 shows the conceptual framework and the research objectives of the study.

## Methods

Based on the findings of other large-scale CQI initiatives [72], this study will be based on a quasi-randomised cluster-controlled trial allowing for a comprehensive and rigorous evaluation of the proposed intervention [73]. Such a design has been used to better understand both processes and outcomes of CQI [51]. Within the province of Quebec, clinics from randomly selected health regions will receive the intervention, while others will serve as controls (no CQI intervention). Intervention clinics will be matched with control clinics. The matching will be based on the clinic level (1 to 10, based on the number of patients registered and services offered) [74]. Clinics in the control group will receive an audit on a selection of AA indicators (Table 1) and will be offered the intervention 12 to 18 months following their recruitment.

**Table 1:** Definition of AA measures

Measure	Definition
**Outcome**	
3rd next available appointment (weekly)	Number of days before the 3rd next available appointment open for general consultation
Relational continuity (monthly)	Total number of medical consultations with a patient’s attached family physician (or specialised nurse) out of the total number of consultations with any family physician (or specialised nurse) from the clinic. Evaluates relational continuity between the provider and their registered patients
**Process**	
48-h capacity (weekly)	Proportion of appointments available in the next 48 h. Provides an overview of the provider’s ability to respond to urgent care demands
Use of walk-in clinics (monthly)	Proportion of consultations with registered patients offered through walk-in visits out of the total number of consultations with their attached professional
Professional diversity of care (monthly)	Proportion of consultations with a physician, resident or specialised nurse out of the total number of consultations with any provider at the clinic. Evaluates the level of involvement of different types of providers (social workers, nurses, pharmacists, etc.) with registered patients
14-day capacity (weekly)	Proportion of appointments available in the next 14 days. Provides an overview of the provider’s ability to respond to routine care and follow-up demands
**Balancing**	
Discontinued care for patients with chronic disease (monthly)	Proportion of registered patients with at least one chronic condition who have not consulted the clinic within the last 12 months

**Fig. 3:**
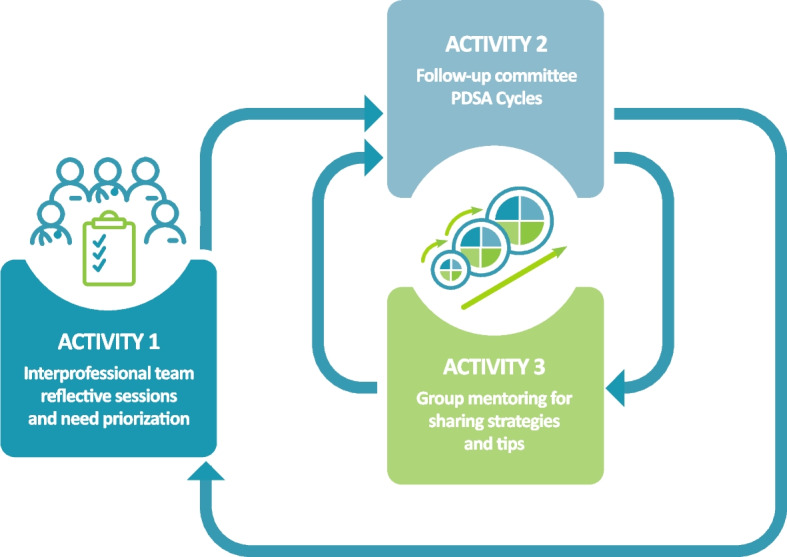
The CQI cohorts

The proposed CQI intervention present in Fig. 3 consists of three activities carried out iteratively until the improvement objectives are achieved or up to a maximum of 18 months of intervention: 1) team reflection and prioritisation of change needs; 2) PDSA cycles; and 3) group mentoring.

### Activity 1: reflective sessions and problem prioritisation

An essential condition for people to become involved in improvement initiatives is that they arrive at a shared understanding of the issues to be solved or the challenges to be overcome, which requires a strong commitment from the participants [20, 75–77]. Participants in the CQI intervention need to *understand the problem and its root causes and contributing factors *[58]. To this end, we will organise clinic-wide meetings labeled *reflective sessions*. These face-to-face sessions, led by the research team and a QI coach, will involve all team members at a given clinic, including clinical, administrative and management staff. During these sessions, we will identify and prioritise AA-related issues. To do so, a summary of AA processes and outcomes will be presented to the group. These will be populated with a comprehensive assessment of both AA processes (using ORAA questionnaires [18]) and outcomes (from the clinic’s electronic medical record [EMR] data and patient-reported experiences of access; see Data collection section below). Through customised facilitation activities, participants will be asked to generate and prioritise ideas to improve AA while considering the concerns of all team members. These activities will maximise engagement of team members and foster discussions and collaboration among them. Examples of facilitated activities include brainstorming sessions, design thinking sessions, root-to-cause analysis and any other activities that might address the specific AA-related issues faced by the team [78]. The end result of Activity 1 consists of an improvement aim [79] focused on a particular AA pillar, identifying the goal(s) the team would like to achieve before the next reflective session, who will benefit from this improvement, how change will be measured and by when.

### Activity 2: PDSA cycles

Following the reflective sessions, we will identify a QI team from each clinic, comprised of at least one team member from all categories of staff (e.g. physicians, nurses, administrative staff, other PHC providers, patients) for a maximum of 5–8 individuals. Each member of the QI team will have the responsibility, through PDSA cycles, to reach the aim(s) determined during Activity 1. Supported by the lead research team, they will convene (every 2 to 4 weeks for approximately 30 min) to review the clinic’s aims (first meeting), then develop (Plan) and implement changes (Do), monitor the effect of these changes on key AA measures (Study) and review the process in order to maintain changes or adjust future actions (Act) [80, 81]. These meetings will take place virtually. More frequent meetings will be organised as needed if the pace of the cycles requires it.

### Activity 3: group mentoring

As teams engage in PDSA cycles, they will test change strategies and generate knowledge about strategies that do or do not work in their specific context [20]. This knowledge will be documented in change packages that include a clear description of the strategy, the indicators used to evaluate its impact, lessons learned and a sustainability plan [60]. These packages have been reported as being highly valuable in supporting organisational change [63, 82]. Group mentoring will aim to increase the effectiveness of the QI coach’s follow-ups and to optimise knowledge transfer of AA improvement strategies among clinics. The first part of the meetings will consist of continued education material about AA- and CQI-related theories and will be provided by the three QI coaches recruited for the trial. Then, participants will split into groups based on their respective AA pillars of focus. A member of each QI team will be invited to share their learnings from the ongoing PSDA cycles (change strategies adopted, lessons learned, indicators used, data extraction algorithms from the EMR). These exchanges will help improve the change packages for each of the AA pillars. These virtual meetings will be held every 6–8 weeks.

Once an improvement aim has been achieved (expected after 3–9 months), the PHC clinic members will reconvene for another reflective session. The QI team will present the changes implemented during the cycles. These sessions will provide a regular meeting place for all team members to voice their concerns and ideas, which can then be used by the QI team during subsequent PDSA cycles. Depending on their new improvement aim (and associated pillar), the QI team will be invited to join an ongoing mentoring group or to form a new one.

### Recruitment

Forty-eight multidisciplinary PHC clinics will be recruited in close collaboration with our key stakeholders using voluntarism and purposeful sampling, with the objective of covering a wide range of organisational structures and contexts, including geographical and socioeconomic differences. To be recruited, clinics must offer interprofessional care (beyond the physician-nurse only model) that is physically located under the same roof. To take part of the study, team member should sign the electronic inform consent form, and at for the PHC clinics at least 50% of all team members should accept to take part in the study to be eligible.

### Sample size justification

The Quebec CQI pilot project showed that the average time to the 3rd next available appointment (primary outcome) is 11 days (sd = 10). Forty-eight clinics (clusters) averaging 15 professionals per clinic for a total of 720 professionals over six measurement time points will be measured every week or month (depending on the measure). An assumed intracluster correlation (ICC) of 0.364 (from the pilot project) provides about 80% power to detect a small effect size of 0.25 (Cohen’s d, assuming that variation will increase over the course of the intervention). This translates into a 50% reduction in time to the 3rd next available appointment from the baseline assessment [83]. Variations in clinic size have been considered (coefficient of variation of 0.49 according to the pilot project) [84]. No inflation for loss to follow-up is planned because the data is readily obtained from the EMR system for all professionals. Provision for one interim analysis 12-months post recruitment is planned using the Demets and Lan’s alpha spending function approach [85]. Thus, we aim to recruit 48 PHC clinics (24 interventions and 24 controls) for the trial proposed in this study.

### Data collection

Complementary qualitative and quantitative data will be collected over a period of 24 months. To evaluate the proposed trial objectives, data collection will be extended by 12 months *in the intervention group only*. We will collect data from all clinics in the intervention group for at least 3 months prior to the start of the intervention.

### Quantitative component

#### Organisational, teamwork and individual context

To document contextual factors (Obj. 1 & 3), the organisational context of the clinic will be assessed using the complete Model Maturity Matrix and the Model for Understanding Success in Quality (MUSIQ) based on the observations of the research team with the collaboration of the clinic’s manager [86, 87]. Then, a self-reported survey composed of a total of 45 items adapted from several questionnaires will be used. Teamwork context will be assessed using the mean score of the Team Climate Inventory short-form (TCI) (19 items) [88, 89]. Job satisfaction will be measured using the Mini Z Burnout Survey (10 items) [90, 91]. Work engagement (3 items) will be measured using the Copenhagen Psychosocial Questionnaire (COPSOQ) [92]. Team composition will be expressed by the number and types of providers working at the clinic, and team stability will be measured by the length of time each team member has been in the clinic as well as the stability of the team throughout the length of the project. To assess the individual context, the Improvement Readiness scale of the SCORE survey [93, 94] (5 items) as well as the agree subscale of the Quality improvement commitment instrument (8 items) will be used [95, 96]. The overall questionnaire should take around 10 to 15 min to complete.

#### AA structural change & processes (Obj. 2)

Each team member will complete the “*Outil Réflexif Accès Adapté*” (ORAA) questionnaire, which provides a comprehensive portrait of the participant’s adherence to each AA pillar [18]. Two versions of the questionnaire are available online in both French and English, one for PHC providers (39 items) and one for administrative staff (25 items), and take approximately 12 min and 6 min to complete, respectively. Each respondent is given a unique identifier to ensure confidentiality through a double validation process. Upon completing the questionnaire, respondents receive a personalised report and suggestions to improve their AA practice. Modifications to AA change packages will also be thoroughly documented as the project evolves.

#### AA Outcomes

Six key AA measures (see Table 1) will be extracted on a weekly or monthly basis via the EMR (Obj. 3). This has been accomplished with great success by the Quebec team over the last two years. Most of these indicators have been used in previous Quebec CQI initiatives on AA. These outcomes will be used to evaluate the effectiveness of the intervention and inform the PDSA cycles (among other indicators chosen by the clinics to document their PDSA cycles). We have successfully automated extraction of the six indicators from an EMR in Quebec. A scale-up process to the other EMRs is ongoing and will be finalised upon trial launch.

To assess effectiveness from patients’ perspectives (Obj. 3), patient-reported experiences will be assessed at each clinic using a 50-item online survey. The questionnaire has been developed based on comparable tools [80, 97–100] and recommendations from our patient committee. The 50 items cover various dimensions of access from a patient perspective, such as the appointment process, communication and team collaboration. The survey is available in French and English and takes approximately 11 min to complete. An electronic informed consent form precedes the survey. The survey will be distributed electronically to all registered patients for whom an email address is available (estimated between 40 and 80% depending on the clinic). This data collection strategy is inexpensive and includes friendly reminders. Both the questionnaire and distribution mode has been tested with success by the Quebec research team. Cognitive testing [101] has been used with eight patients to validate the tool.

### Qualitative component

Document analysis and non-participant observations [102–104] will be collected to document and compare structural organisational changes and processes of care with respect to AA (Obj. 2). All documents produced by the teams (e.g. PDSA cycle journaling, change packages) and all meeting minutes related to the three activities of the intervention will be summarised per clinic by the QI coaches using a coding grid. In addition, a logbook will be completed each week to document completed tasks and time spent on them. Every 3 months, QI coaches will evaluate the progress of each clinic for which they are responsible using the Assessment Scale for Collaboratives [105].

At the end of the intervention (T18), as well as 12 months after the intervention (T30), we will conduct focus groups with purposefully selected members of the QI teams to document their ongoing perspectives on CQI processes, their appreciation of CQI methods and the perceived impact on their practice (Obj. 1). Implementation issues and the need for additional training or educational tools will be discussed. With the intervention group, we plan to conduct an average of 10–12 interviews at three times (T12, T18, T30) to better understand the level of appreciation for QI and barriers and facilitators to engaging in QI activities. We will interview a variety of professions and roles within the clinic to ensure information-rich discussions [106].

### Data analyses

For the first objective, the analysis will be based on the InQuIRe framework (Fig. 2). Audio recordings and transcripts of interviews will be reviewed simultaneously, to assess validity of the transcription process, and analysed using an iterative approach [107, 108]. Thus, the analysis process will progress as follows throughout data collection for each site: 1) development of a mixed deductive-inductive classification grid; 2) coding according to the classification grid of an initial interview; 3) discussion and team consensus on the final grid; 4) linking of the various emerging themes; 5) coding of all interviews; and 6) validation by two judges (counter-coding) of at least 20% of the material in order to validate the classification. Data saturation will be determined as defined by Constantinou et al. [109] This will be followed by an inter-site analysis to identify commonalities between and specificities of the sites examined [110] and to compare the different configurations between sites [111]. Data will be analysed by wave first. Finally, matrices based on waves will be generated to identify particular intra- and inter-wave patterns [112].

Strategies used to instill structural changes in the organisation and processes of care (Obj. 2) will be rigorously documented and categorised by AA pillar. During PDSA cycles of a given strategy, its efficacy within each clinic will be assessed using run charts [113] and control charts [114] and their respective rules of interpretation. In addition to distinguishing systematic changes from chance variation [115], these types of graphs will allow for monitoring of the performance of the strategy developed by the clinics to improve AA and inform the PDSA cycles (Activity 2 of the intervention).

The impact of the intervention on AA outcomes (Obj. 3) will be structured around the quintuple aim [116]. For all outcomes, we will describe clinics and participants using means and standard deviations or medians and interquartile ranges. Also, time series analyses will be performed based on EMR indicators to detect changes since baseline, at the beginning of the intervention (T3), during the intervention (T3–T18) and the sustainability of the changes (T30). *Care team functioning*: To account for clustering of providers within clinics, the primary outcome measure, 3rd next available appointment, will be analysed using a generalised linear mixed model (with log link and quasi-Poisson distribution) adjusting for time to account for secular trends as well as important contextual factors such as number of providers and readiness for change. We will inspect missing data, and if more than 10% of the sample has missing values, we will use multiple imputation techniques. Otherwise, the maximum-likelihood method of estimation is considered adequate to address missing data in such regression models [117, 118]. Differences between the control and intervention periods and time trends will be estimated using odds ratios and their 95% confidence intervals. Similar regression models will be used (as dictated by the distribution of the data) to assess differences in % of 48-h open slots, walk-in usage, relational continuity and multidisciplinary involvement. *Patient experience*: A comprehensive description of patients’ experiences will be generated three times over the study. Items that focus on ease of access, perception of interprofessional collaboration, satisfaction with the appointment process and consultations outside the clinic for non-urgent care will be used for longitudinal analysis purposes. *Population health*: Discontinuity for chronic patients will be analysed using a generalised linear mixed model to assess the impact of the project on the management of chronic patients. *Health equity and inclusion*: Accessibility and patient experience will be stratified by visible minority status, ethnicity, preferred language, gender and physical or mental ability. If any disparity seems apparent, this issue will be addressed during the improvement efforts. *Efficiency:* Total costs to the system related to the implementation of the intervention as well as costs per clinic and cohort, accounting for different organisational characteristics, will be calculated. We will analyse the QI coach’s logbook to assign costs to the different tasks performed. Incremental ratios (i.e. change in costs to use the intervention divided by change in the 3rd next available appointment) will also be calculated.

The AA process and outcomes of the intervention (Obj. 4) will be documented 12 months after the end of the intervention to assess the sustainability of its effects over time. Regression models similar to those used in objective 3 will be used to identify the factors that influence sustainability of the intervention effects. Factors such as team context, participation in each intervention activity, support duration (wave) and progress made during the intervention will be included in the model. The association between the sustainability of outcomes and each factor will be estimated using regression estimates and their 95% confidence intervals. We will also evaluate whether the practices put in place (processes) are maintained.

In addition to the analyses described above, one interim analysis will be conducted after 12 months of the intervention. This analysis will focus mainly on the trial’s primary outcome (3rd next available appointment) to identify early evidence of the intervention’s superiority. This study will be monitored by an independent Data Safety and Monitoring Committee (DSMC) formed by the steering committee. The DSMB will consist of an independent healthcare organization expert, a PHC physician and a statistician who are not involved in this study. The interim analysis will be performed by a statistician who is blind to the treatment allocation. Members of the DSMC will also be responsible for developing terms of reference, including stopping rules to be used during interim analysis. The steering committee will be notified by the DSMC only if the latter considers continuance of the study to be futile.

## Discussion

The goal of this trial is to improve access to PHC for patients. Thus, this research will have a direct impact on patient experiences of care. Also, this study will develop PHC team capacities through PDSA cycles and large-scale CQI group mentoring. It will be a powerful driver for providers to engage in CQI initiatives with their teams to address timeliness, accessibility, continuity and interprofessional collaboration. We also hope to generate unique opportunities to engage providers in meaningful organisational change based on their local needs and priorities [119] while using readily accessible information from the clinic EMR [34]. Ultimately, the study will also put in place the structure and expertise necessary to allow for the intervention to spread across the province.

To the best of our knowledge, this is the first prospective controlled trial that proposes to study an externally facilitated CQI intervention to improve timely access. Our mixed-method approach offers a unique opportunity to contribute to the scientific literature on large-scale CQI cohorts to better understand the processes of a rigorously implemented CQI intervention [120]. This research will contribute to unpacking the process of facilitating change within a PHC clinic, thereby filling the gap on how to generalize CQI interventions [121, 122] and accelerate the spread of effective AA improvement strategies while strengthening local QI culture within clinics [51]. Our study will also provide some insights on the contextual factors and CQI processes that contribute to the sustainability of an intervention aiming to improve access in PHC.

We expect to face two main issues that may limit the impact of the intervention’s effectiveness. First, we cannot overlook resistance to change and clinician commitment to this longitudinal intervention. We believe, however, that the socio-political context in which PHC professionals operate, where access is a growing priority of governing bodies, will help maximize professional engagement and sustain interest. Our bottom-up approach, which is based on CQI techniques rather than performance achievement pursuits and is tailored to each clinic's contextual reality, will also help maintain the professionals’ motivation to change and stay involved in the initiative. Finally, as observed during the pilot project [19], our pragmatic approach, in which data collection to achieve scientific objectives is almost entirely embedded in daily clinical activities (EMR), will ensure that participants quickly see the impact of their involvement in the project without the need for tedious form completion or data extraction processes.

Secondly, the standardization and fidelity of the CQI approach is crucial to enable the production of evidence on the effectiveness of the intervention and allow for its generalization on a larger scale. To do this, we have set up a rigorous journaling process for the four coaches who will be involved in the project for the purposes of monitoring the intervention and quickly bringing modifications if necessary. Team meetings (coaches and the two principal investigators) inspired by medical rounds have also been planned to share the evolution of the change strategies implemented in each clinic and to promote knowledge sharing between the coaches.

## Conclusion

This cluster-controlled trial research project based on a mixed-method approach offers a unique opportunity to contribute to the scientific literature on large-scale CQI cohorts to improve AA in primary care teams and to better understand the processes of CQI.

## Data Availability

Not applicable.
